# The Lunacy Laws

**Published:** 1901-09-14

**Authors:** 


### THE LUNACY LAWS.

A case came before the Richmond Police Court last week which serves to show how rigidly the law in regard to the reception and care of lunatics is being enforced. The defendants were charged with receiving and taking charge of lunatics in an uncertified institution, and Mr. Guy Stevenson, who appeared for the prosecution, laid it down that provided the persons referred to were shown to be lunatics the honest belief of the defendants that th ey were sane was not material to the issue, which without doubt is the letter of the law on the subject. He did not seek to show that the defendants knew they were lunatics, nor was there any suggestion of improper treatment or want of sufficient care of the patients. Three medical practitioners who had attended one or other of the patients in question stated that they did not consider them to be certifiably insane, and it was shown that one case had been taken to an eminent specialist in mental cases who had refused to certify. On the other hand two lunacy experts were called on behalf of the prosecution, the Lunacy Commissioners, according to whom the patients were suffering from general paralysis "which necessarily involved dementia." According to the *Richmond Herald* Dr. Hugh Gordon Hill gave evidence that these cases were suffering from general paralysis, and in cross-examination he laid it down that "general paralysis was the most marked form of insanity, and persons suffering from it *must be lunatics*." Dr. Henry Rayner, of St. Thomas's Hospital, was a little more cautious, and admitted that "doctors differed as to the degree of general paralysis at which a case was certifiable." It is worth noting, however, that if the law is to be rigidly applied the question of a lunatic being certifiable does not come up. If he is a lunatic he must not be received except under certificate—quite independent of the fact that in some cases such a certificate cannot be obtained. It is there that the hardship lies. In the end one case was dropped, and in regard to the others the Bench imposed fines and costs amounting to £58 9s. 3d. That the administration of the Lunacy Laws according to their strict letter may lead and does produce hard cases goes without saying. That is a question we need not touch, nor do we say anything as to the merits of the cases in question, for indeed by the time the inspection was made they were pretty obvious. It is significant, however, that even then one case was withdrawn. We merely point out that the magistrates were asked to decide the case according to the rigid letter of the law, and what we would urgently insist upon is that such an administration of the Lunacy Acts makes it next door to impossible for incipient cases of insanity to receive proper treatment, for until the case has arrived at such a stage as to be "certifiable" no one who has anything to lose will touch it. The whole trouble arises from the fact that while the law draws no distinction and deals with the whole class of lunatics *en masse*, the machinery for their control deals solely with one section of it, namely, those persons who are "certifiably insane." Between the limits of these two definitions there lies a large field, including within it most cases of incipient lunacy and many of those lunatics at large who are such a nuisance and such a danger to the community. Not only is no provision made for these people, but heavy penalties are inflicted on anyone who in the words of the Act "for payment takes charge of, receives to board or lodge, or detains" them in an unlicensed house; while into a licensed house they cannot get until they become "certifiable." It is idle for us to bewail the dangers arising from "the lunatic at large' while we penalise every effort to take care of him.

